# Correction to “Transition Shock Among Nursing Interns: Roles of Achievement Motivation and Clinical Practice Environment—A Cross‐Sectional Study”

**DOI:** 10.1002/nop2.70472

**Published:** 2026-11-03

Chen S, Liu W, Li Y, et al. 2025. “Transition Shock Among Nursing Interns: Roles of Achievement Motivation and Clinical Practice Environment—A Cross‐Sectional Study.” *Nursing Open* 12, no. 9: e70297.

In the originally published version of this article, there was an error in the Funding information section. Although the funding agency was correctly stated as Medical Science and Technology Foundation of Guangdong Province, the funding project number was incorrect.

Incorrect:

20211124204928

Correct:

B2022339

We apologize for this error.

